# Imbalance of Wnt/Dkk Negative Feedback Promotes Persistent Activation of Pancreatic Stellate Cells in Chronic Pancreatitis

**DOI:** 10.1371/journal.pone.0095145

**Published:** 2014-04-18

**Authors:** Yanling Hu, Rong Wan, Ge Yu, Jie Shen, Jianbo Ni, Guojian Yin, Miao Xing, Congying Chen, Yuting Fan, Wenqin Xiao, Gang Xu, Xingpeng Wang, Guoyong Hu

**Affiliations:** 1 Department of Gastroenterology, Shanghai Tenth People's Hospital, Tongji University School of Medicine, Shanghai, China; 2 Department of Gastroenterology, Shanghai First People's Hospital, Shanghai Jiaotong University School of Medicine, Shanghai, China; Centro Nacional de Investigaciones Oncológicas (CNIO), Spain

## Abstract

The role of persistent activation of pancreatic stellate cells (PSCs) in the fibrosis associated with chronic pancreatitis (CP) is increasingly being recognized. Recent studies have shown that Wnt signaling is involved in the development of fibrosis in multiple organs, however, the role of specific Wnts in pancreatic fibrosis remains unknown. We investigated the role of Wnt signaling during PSC activation in CP and the effect of β-catenin inhibition and Dickkopf-related protein 1 (Dkk1) restoration on the phenotype of PSCs. CP was induced in mice by repetitive caerulein injection and mouse PSCs were isolated and activated *in vitro*. The expression of Wnts, β-catenin, secreted frizzled-related proteins (sFRPs) and Dkks was analyzed by quantitative RT-PCR and western blotting. The canonical Wnt signaling pathway was examined by immunofluorescence and western blot detection of nuclear β-catenin expression. The effect of recombinant mouse Dkk-1 (rmDkk-1) on cell proliferation and apoptosis was assessed by flow cytometry, immunofluorescence, immunocytochemistry and Cell Counting Kit-8 (CCK-8) analysis. The expression of β-catenin, collagen1α1, TGFβRII, PDGFRβ and α-SMA in PSCs treated with different concentrations of rmDkk-1 or siRNA against β-catenin was determined by quantitative RT-PCR and western blotting. Wnt2 was the only Wnt whose expression was significantly upregulated in response to PSC activation, and Wnt2 and β-catenin protein levels were significantly increased in the pancreas of CP mice, whereas Dkk-1 expression was evidently decreased. Nuclear β-catenin levels were markedly increased in activated PSCs, and rmDkk-1 suppressed the nuclear translocation of β-catenin and the proliferation and extracellular matrix production of PSCs through the downregulation of PDGFRβ and TGFβRII. Upregulation of Dkk-1 expression increased apoptosis in cultured PSCs. These results indicate that Wnt signaling may mediate the profibrotic effect of PSC activation, and Wnt2/Dkk-1 could be potential therapeutic targets for CP.

## Introduction

Chronic pancreatitis (CP) is characterized by progressive pancreatic fibrosis. The persistent activation of pancreatic stellate cells (PSCs) plays a pivotal role in pancreatic fibrogenesis, while the redifferentiation and outcome of activated PSCs determines the pathological outcome after pancreatic injury [1]–[3]. The transient activation of PSCs during acute pancreatitis suggests that a negative feedback mechanism regulates PSC activation [4]. In addition, studies have shown that restoration of quiescence in activated PSCs may provide a novel therapeutic strategy for CP [5], [6].

Although several inflammatory mediators released during the development of pancreatitis have the potential to regulate PSCs, accumulating evidence supports major roles for platelet-derived growth factor (PDGF), transforming growth factor (TGF)-β1 and angiotensin II as key modulators of the persistently activated and profibrotic phenotype of these cells [7]. PDGF can induce the proliferation of PSCs and contributes to the migratory potential of PSCs, whereas TGF-β1 and angiotensin II can induce PSCs to express α-smooth muscle actin (SMA) and extracellular matrix (ECM) proteins [7]. Although the mechanism of PSC activation has been examined in recent studies [4], the precise molecular mechanisms underlying the persistent activation process remain to be elucidated.

The Wnt signaling pathway is highly conserved in multicellular organisms and regulates cell fate in development and disease [8], [9]. Two Wnt signaling pathways have been described, canonical Wnt signaling and noncanonical Wnt signaling. Activation of the canonical pathway results in the accumulation and nuclear translocation of β-catenin. As a key target of the canonical Wnt pathway, β-catenin mediates many cellular processes in response to Wnt [10], [11]. The Dickkopf protein families (Dkks) are well-known Wnt antagonists that negatively modulate the canonical Wnt pathway [12], [13]. Several recent reports demonstrated that Wnt signaling plays a role in fibrosis development in multiple organs [14]–[18]. Furthermore, the involvement of functional Wnt signaling in activated hepatic stellate cells (HSCs) in liver fibrosis has been demonstrated [19], [20]. Since PSCs and HSCs share many morphological and functional features [1], [21], we hypothesized that the activation of PSCs may be dependent on Wnt signaling activation and the imbalance of Wnt/Dkk negative feedback may promote the persistent activation of PSCs in CP. To test this hypothesis, we investigated the role of Wnt signaling and Dkk in PSC activation *in vivo* and *in vitro*. Our findings showed that the canonical Wnt signaling is involved in the activation of PSCs in CP. Furthermore, the Wnt signaling antagonist Dkk1 suppressed the proliferative and profibrotic phenotype of PSCs by downregulating the expression of collagen1α1, TGFβRII and PDGFRβ through the inhibition of Wnt/β-catenin signaling.

## Materials and Methods

### Ethics statement

All the animal related procedures were approved by the Animal Care and Use Committee of The Tenth People's Hospital of Shanghai(permit number: 2011-RES1). This study was also approved by Science and Technology Commission of Shanghai Municipality (ID: SYXK 2007-0006). The mice were kept at 18°C–26°C on a 12 h light and dark cycle with free access to water and standard mice chow. They were allowed to acclimatize for a minimum of 1 week. The environment was maintained at a relative humidity of 30%–70%.

### Animals and reagents

Male Balb/c mice were purchased from Shanghai SLAC Laboratory Animal Co., Ltd. (Shanghai, China). Trizol reagent was from Invitrogen (Carlsbad, CA, USA). The RT reagent kit and Premix Ex Taq were from TAKARA (Kusatsu, Japan). Caerulein, Hoechst 33342, propidium iodide (PI), Cell Counting Kit-8 (CCK-8), 4′,6-diamidino-2-phenylindole (DAPI) and the antibody against bromodeoxyuridine (BrdU) were purchased from Sigma Chemical (Sigma-Aldrich, St. Louis, MO, USA). The antibody against β-catenin was from Cell Signaling (CST, Danvers, MA, USA). The antibody against Dickkopf-1(Dkk-1) was from Abcam (Abcam, Cambridge, MA, USA). Antibodies against desmin, α-smooth muscle actin (α-SMA), Ki-67 and Lamin A were from Santa Cruz Biotechnology (Santa Cruz, CA, USA). The antibodies against Wnt2 and glyceraldehydes-3-phosphate dehydrogenase (GAPDH) were from Epitomics (Burlingame, CA, USA), and HRP-conjugated secondary antibodies were from Jackson ImmunoResearch (West Grove, PA, USA). Recombinant mouse Dkk-1 was from R&D Systems (Minneapolis, MN, USA). Unless otherwise stated, all other chemicals were purchased from Sigma and cell culture reagents were from Gibco-BRL.

### Induction of chronic pancreatitis in mice

All animal experiments were approved by the Animal Care and Use Committee of Tongji University. Chronic pancreatitis was induced in mice by repetitive acute pancreatitis as previously described [22], [23]. Acute pancreatitis was elicited by hourly (six times) intraperitoneal injections of 50 µg/kg body weight caerulein (Sigma-Aldrich, St. Louis, MO, USA), whereas control animals received a comparable amount of normal saline (NS). Mice were subjected to three episodes of acute pancreatitis per week for 4 weeks. Mice were sacrificed 4 weeks after the first caerulein injection under anesthesia with 3% pentobarbital sodium and pancreatic tissue samples were collected.

### Isolation, culture of mice PSCs and rmDkk-1 treatment

Primary PSCs were isolated from normal male Balb/c mice by digestion of pancreatic tissue and Nycodenz density gradient centrifugation [5], [24]. Freshly isolated mice PSCs were cultivated in DMEM/F12 supplemented with 10% FBS and 1% penicillin–streptomycin (Gibco BRL, USA) at 37°C, 5% CO_2_. On the second day and every day thereafter, the culture medium was changed and treated with or without rmDkk-1 at different doses as described in the relevant figures.

### Immunofluorescence

The phenotype of PSCs was evaluated by immunofluorescence using antibodies specific for mouse desmin and α-SMA. PSCs were probed against a primary monoclonal anti-Ki-67 antibody to examine Ki-67 reactivity in addition to anti-β-catenin and anti-Wnt2 antibodies. For desmin, the staining sequence was goat-anti-mouse desmin (1∶50) and rhodamine-labeled anti-goat (1∶800). The staining sequence for α-SMA was mouse-anti-mouse α-SMA (1∶100) and rhodamine-labeled anti-mouse (1∶800). The staining sequence for Ki-67 was goat-anti-mouse Ki-67 (1∶50), and rhodamine-labeled anti-goat (1∶800). Rabbit-anti-mouse β-catenin (1∶100) and rabbit-anti-mouse Wnt2 (1∶100) were also used for immunofluorescence. Nuclear counterstaining was performed using DAPI, and staining was observed by confocal microscopy (Zeiss, LSM710).

### Quantitative reverse transcriptase-PCR (qRT-PCR)

PSC mRNA levels were detected by qRT-PCR for desmin, α-SMA, β-catenin, PDGFRβ, TGFβRII, Collagen1α1, Wnts, Dickkopf (Dkk) and secreted frizzled-related protein (sFRP) family. Briefly, total cellular RNA was extracted from PSCs using the Trizol reagent (Invitrogen, Carlsbad, CA, USA) and then quantified. RT reactions were performed with total RNA (2 µg) according to the ExScript RT reagent kit. Real-time PCR was performed in triplicate for each gene of interest under each triplicate experimental condition using the ABI Prism 7900HT Sequence Detection System (Applied Biosystems, CA, USA). GAPDH was used as a separate endogenous control to which each gene of interest was normalized. Fold changes and subsequent percentage gene expression levels relative to the designated control groups were calculated using the comparative CT (2^−ΔΔCT^) method [25]. The primer sequences are shown in Table 1.

**Table 1:** Primer Sequences Used for qRT-PCR Analysis.

Gene		Primer sequence (5'→3')
Desmin	Forward	TCTCTGAGGCTGAAGAATGGT
	Reverse	TCAATCTCGCAGGTGTAGGA
α-SMA	Forward	TGCCGAGCGTGAGATTGT
	Reverse	CCCGTCAGGCAGTTCGTAG
β-catenin	Forward	AGGGTGCTATTCCACGACTA
	Reverse	CACCCTTCTACTATCTCCTCCAT
Dkk-1	Forward	TGAGGGCGGGAACAAGTA
	Reverse	TTCGGCAAGCCAGACAGA
Dkk-2	Forward	ATGATGGAAACCTGGATTGGAA
	Reverse	GAGGCACATAACGGAAGCA
Dkk-3	Forward	GGCAAGGGAATGTGGTAGAG
	Reverse	GTTGGGCGTTTGATTGTAGC
Dkk-4	Forward	AGCACATAAATAAAGCCCAATCTC
	Reverse	ATAGTAGACGCAAAGCCAAAAGT
sFRP-1	Forward	TCTTCCTCTGTTCGCTCTT
	Reverse	GGCTTCCGTGGTATTGG
sFRP-2	Forward	GTCATGTCCGCCTTCG
	Reverse	TCGTCCTCATTCTTGGTTTT
sFRP-4	Forward	TTTGTCACCTATCCCTCG
	Reverse	CCACTGTATGGACCTTCTAC
sFRP-5	Forward	GGGGACCGAAAGTTGATT
	Reverse	GCCCGTCAGGTTGTCTA
Wnt1	Forward	GCCCGCCTCCAGACTTATT
	Reverse	GTTCTGTGCTGCGGTTCT
Wnt2	Forward	CGACACCCAGATGTGATGC
	Reverse	GGCCGATTCCCGACTACTT
Wnt3	Forward	CCCTGCTCTGGATGGTGTA
	Reverse	GCCTGTTCTGTTGCGGTAG
Wnt3a	Forward	AATTTGGAGGAATGGTCTCTCGG
	Reverse	CAGCAGGTCTTCACTTCACAG
Wnt7a	Forward	GGCTTCTCTTCGGTGGTAGC
	Reverse	TGAAACTGACACTCGTCCAGG
Wnt8a	Forward	TTCGCAGGAGTGAAATCGG
	Reverse	AGCTGGGTGGAACGGTAT
Wnt8b	Forward	AATCACCCACATAAACCTTCCG
	Reverse	TCTTCAGTTCTCCCAATACCCAT
Wnt10b	Forward	GAAGGGTAGTGGTGAGCAAGA
	Reverse	GGTTACAGCCACCCCATTCC
Wnt4	Forward	AGACGTGCGAGAAACTCAAAG
	Reverse	GGAACTGGTATTGGCACTCCT
Wnt5a	Forward	AGTCCTTTGAGATGGGTGGTA
	Reverse	CCTCTGGGTTAGGGAGTGTC
Wnt11	Forward	CAGCCTTCGTGTATGCCCT
	Reverse	CCGTAGCTGAGGTTGTCCG
PDGFRβ	Forward	CCAGAAGTAGCGAGAAGC
	Reverse	ATCACCGTATCGGCAGTA
TGFβRII	Forward	TTTCGGAAGAATACACCAC
	Reverse	GACACGGTAGCAGTAGAA
Collagen1α1	Forward	CGCCATCAAGGTCTACTG
	Reverse	ACGGGAATCCATCGGTC

### Western blot analysis

Total proteins were prepared by standard procedures and quantified by the BCA method. An aliquot of 60 µg of protein per sample was loaded onto a 10% SDS-polyacrylamide gel. After electrophoresis, proteins were transferred onto PVDF membranes by electroelution. The membranes were incubated with anti-desmin antibody (1∶200), anti-α-SMA antibody (1∶200), anti-β-catenin antibody (1∶1000), anti-Wnt2 antibody (1∶1000) and anti-Dkk-1 antibody (1∶200) overnight at 4°C. The next day, membranes were washed and incubated with HRP-conjugated mouse-anti-goat IgG (1∶2000), HRP-conjugated goat anti-mouse IgG (1∶2000), and HRP-conjugated goat anti-rabbit IgG (1∶2000) for 1 h at room temperature. After washing, the membranes were developed using the ECL-detection system (Santa Cruz Biotechnology, Santa Cruz, CA, USA), quickly dried, and exposed to ECL film.

### Apoptosis Analyses using Flow Cytometry and Hoechst 33342 staining

Freshly isolated mouse PSCs were seeded at a density of 2×10^6^ cells in 25 cm^2^ coated plastic bottles and cultured as previously described. On day 5, cells were collected, washed twice in cold PBS, mixed in 100 µl of 1× binding buffer, and incubated at room temperature for 15 min with an annexin-V/PI (BD Biosciences) double staining solution. Stained cells were analyzed by flow cytometry, and the percentage of apoptotic cells was calculated using ModFitLT software (Verity Software House).

Apoptosis in PSCs was determined using Hoechst 33342 staining. Briefly, cells were stained with 8 mg/ml Hoechst 33342 and examined directly by fluorescence microscopy. Cells with nuclei containing condensed and/or fragmented chromatin were considered to be apoptotic [26]. Viable cells (Hoechst negative/dark blue) and apoptotic cells (Hoechst positive/light blue) were counted in 10 different fields at ×200 magnification in each well in three independent experiments by two persons.

### Cell proliferation

Freshly isolated mouse PSCs were seeded at a density of 2×10^5^ cells per well in 24-well plates in DMEM/F12 supplemented with 10% FBS and 1% penicillin–streptomycin. After 24 h, the cells were treated as indicated and incubated for 3 days. Proliferation was determined by the Cell Counting Kit-8 according to the manufacturer's instructions.

For BrdU incorporation assay, 30 mM BrdU (Sigma-Aldrich, St. Louis, MO, USA) was added to the culture medium for incorporation into the DNA of replicating cells. After 2 h of incubation, cells were fixed in 4% paraformaldehyde. Then cells were immunostained for BrdU according to the manufacturer's instructions. Finally, the proliferative cells were counted in 10 different fields at ×200 magnification in three independent experiments by two persons.

### PSC transfection

RNA interference was performed to silence β-catenin expression. PSCs at 1 day were incubated in Opti-MEM Reduced Serum Medium, transferred to a six-well culture plate until reaching 60%–80% confluence, and transfected with 80 nM siRNA negative control or β-catenin specific FAM-siRNA for 6 h using Lipofectamine 2000 (Invitrogen, Carlsbad, CA, USA). Cells were then transferred to DMEM/F12 medium containing 10% FBS without antibiotics for 72 h. Pre-designed siRNAs targeting the coding region of the β-catenin gene (siRNA ID# 9371, # 9558, # 9370) (Shanghai GenePharma Co., Ltd, China) and silencer select negative control siRNA (GenePharma) were used in this study. The sequences are as follows: #9371-5′>3′GCCUCUGAUAAAGGCAACUTT, #9558-5′>3′ GCCUUAGUAAACAUAAUGATT, #9370-5′>3′GCACCAUGCAGAAUACAAATT, negative control-5′>3′ UUC UCC GAA CGU GUC ACG UTT. Specific silencing of the targeted gene was confirmed by reverse transcription polymerase chain reaction (RT-PCR) and western blot analysis. Cells were harvested and used for experiments as described below.

### Statistical analysis

All results were expressed as mean ± standard deviation (SD). Statistical analysis was performed using Student's t-test for comparison of two groups, and ANOVA for multiple comparisons. In both cases, differences of P<0.05 were considered statistically significant.

## Results

### Culture and identification of mouse PSCs in vitro

**Figure 1:**
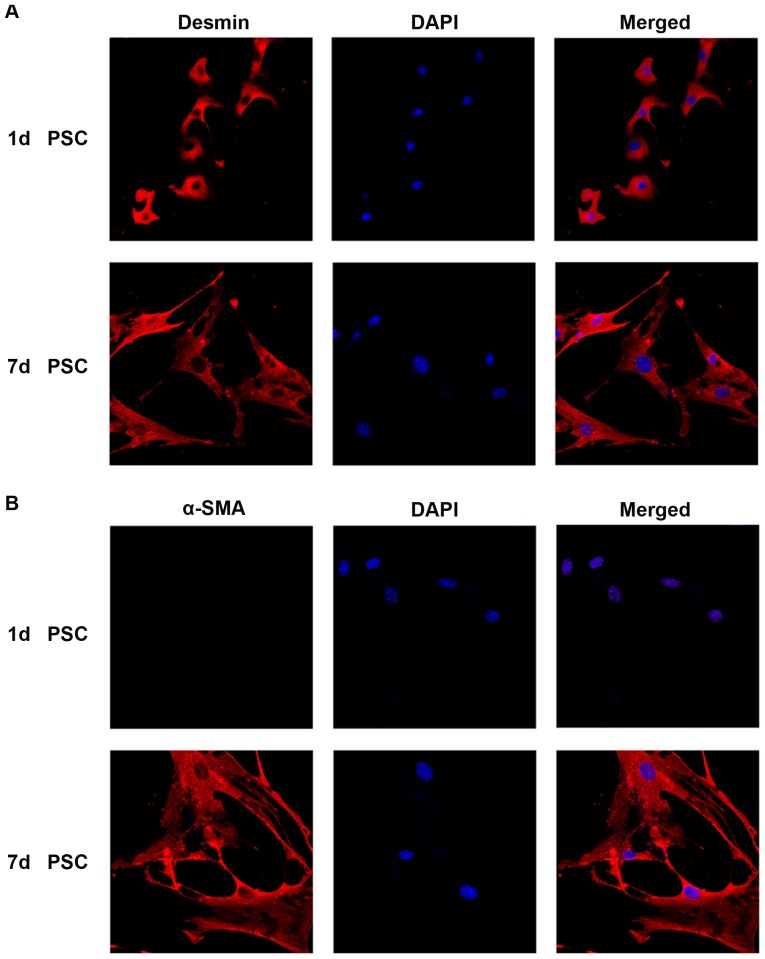
Mouse PSCs activation *in vitro*. Immunofluorescence analysis of desmin and α-SMA during the activation of PSCs (A) Immunofluorescence staining of desmin in day 1 (quiescent) and day 7 (fully activated) PSCs. (B) Immunofluorescence staining of α-SMA in day 1 (quiescent) and day 7 (fully activated) PSCs. DAPI (blue) was used to counterstain nuclei (magnification ×400).

**Figure 2:**
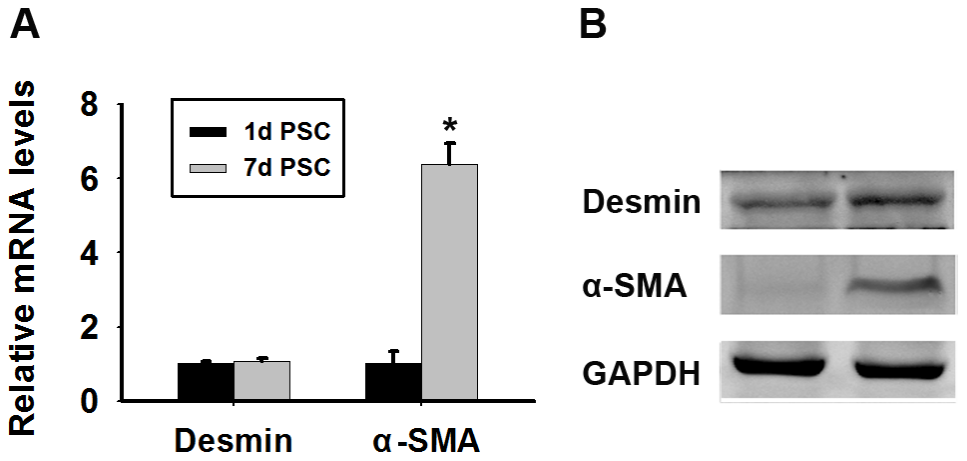
Expression of desmin and α-SMA during the activation of PSCs *in vitro*. (A) Quantitative RT-PCR analysis of the mRNA levels of desmin and α-SMA in PSCs. GAPDH mRNA was used as a housekeeping control. Data are presented as mean±SD from three independent experiments. *p<0.05 compared with quiescent PSCs. (B) Western blot analysis of desmin and α-SMA protein expression in PSCs.

Approximately 1×10^6^ PSCs were harvested from four mice. Freshly isolated PSCs retained a quiescent phenotype for 1–2 days. After 48 h, they began to be activated and became fully activated by day 7 [27]. These cells were identified by real time RT-PCR, western blot analysis and immunofluorescence for desmin and α-SMA. Immunofluorescence analysis showed that more than 95% of isolated cells were desmin-positive (the biomarker for both quiescent and activated PSCs) (Figure 1A), whereas α-SMA was expressed only in activated PSCs (Figure 1B). Both the qRT-PCR (Figure 2A) and western blot analysis (Figure 2B) of α-SMA expression demonstrated the activated phenotype of PSCs.

### The Wnt/β-catenin pathway is activated during the activation of mouse PSCs *in vitro*

**Figure 3:**
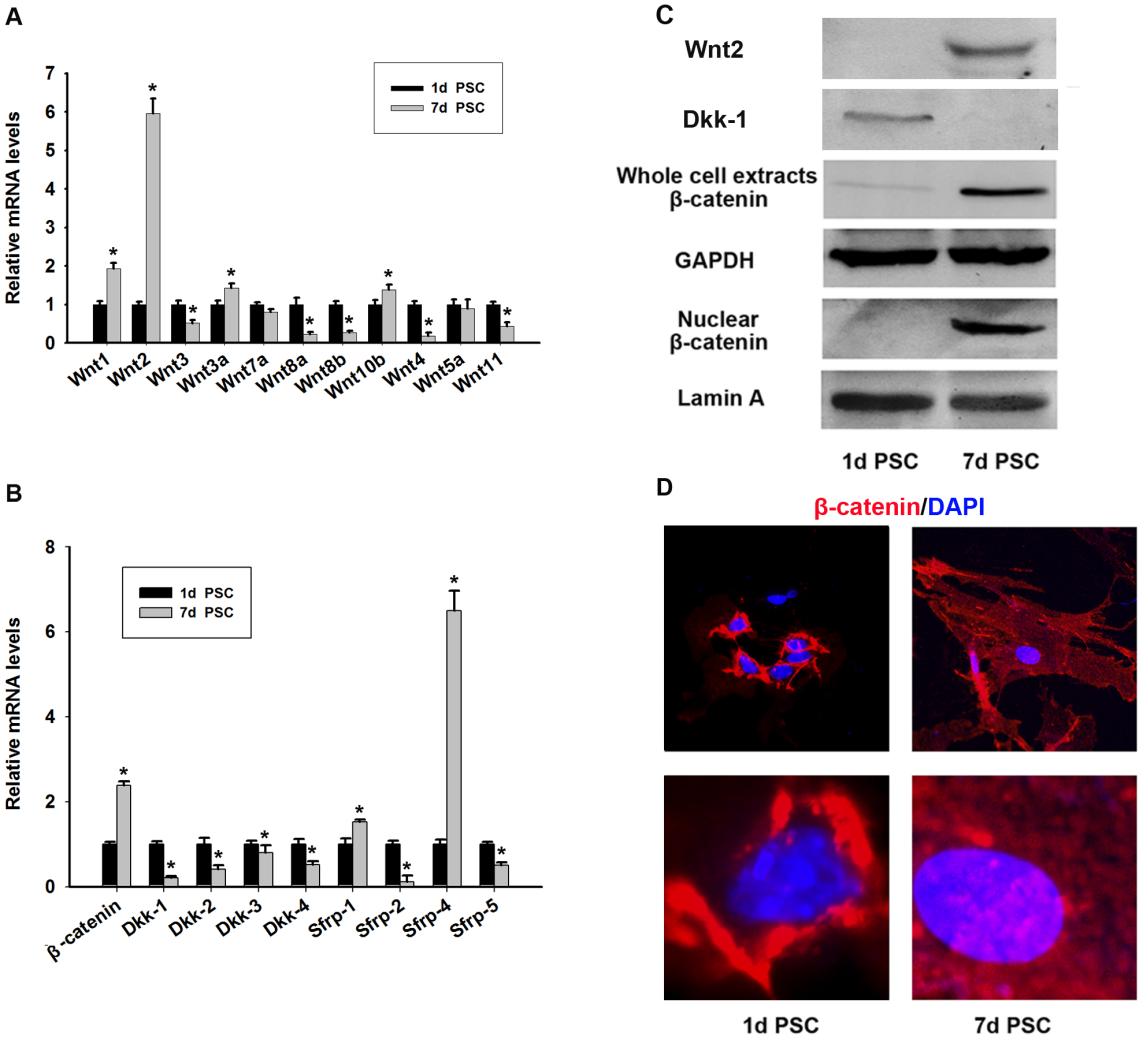
Expression of Wnt/β-catenin, Dkks and sFRPs families during activation of PSCs *in vitro*. Quantitative RT-PCR detection of Wnt1, 2, 3, 3a, 4, 5a, 7a, 8a, 8b, 10b, 11 (A) and β-catenin and Dkk-1, 2, 3, 4 and sFRP-1, 2, 4, 5 (B) in quiescent and activated PSCs. Data are presented as mean±SD from three independent experiments. *p<0.05 compared with quiescent PSCs. (C) The protein levels of Wnt 2, Dkk-1 and β-catenin in quiescent and activated PSCs were detected by western blotting. (D) Immunofluorescent staining of β-catenin (red) in PSCs. DAPI (blue) was used to counterstain nuclei. Nuclear translocation of β-catenin in activated PSCs was observed.

Assessment of Wnt gene expression in day 1 (quiescent) and day 7 (fully activated) PSCs by qRT-PCR showed that Wnt2 was notably increased in day 7 PSCs compared with day 1 PSCs (Figure 3A). Western blot analysis also showed a marked upregulation of Wnt2 expression during the activation of PSCs (Figure 3C). Assessment of the mRNA level of Dkk and sFRP family showed the downregulation of Dkk expression, especially Dkk-1, in day 7 PSCs (Figure 3B). As shown in Figure 3B, the mRNA levels of sFRP1 and sFRP4 were increased while those of sFRP2 and sFRP5 were decreased in day 7 PSCs compared with day 1 PSCs. Western blot analysis confirmed that Dkk-1 was downregulated in day 7 PSCs (Figure 3C). Since the Wnt signaling pathway induces the stabilization and nuclear translocation of β-catenin, we examined the expression of β-catenin in day 1 and day 7 PSCs. A marked increase in the nuclear level of β-catenin was detected in day 7 PSCs, and β-catenin levels were also increased in whole cell extracts (Figure 3C). qRT-PCR showed an increase in β-catenin mRNA levels in day 7 PSCs as well (Figure 3B). Immunofluorescence analysis was performed to confirm the accumulation of nuclear β-catenin, which showed positive staining for β-catenin along the plasma membrane in day 1 PSCs and an increase in nuclear β-catenin staining in day 7 PSCs (Figure 3D). These results suggested that the Wnt/β-catenin pathway was activated during the activation of PSCs.

### Wnt/β-catenin pathway is activated in CP

**Figure 4:**
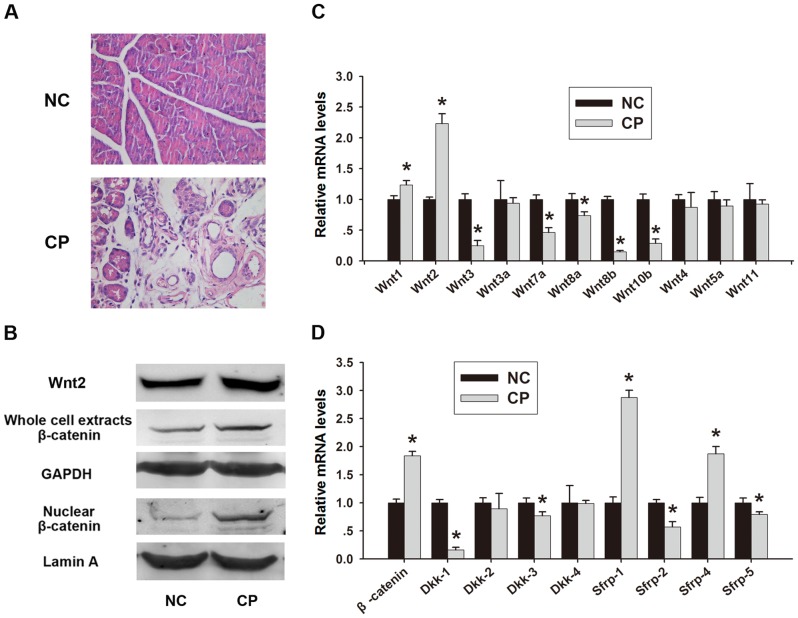
The Wnt/β-catenin pathway is activated in chronic pancreatitis. (A) Histological observations in H&E stained sections (magnification ×400). (B) Western blot analysis of Wnt2 and β-catenin protein expression in the pancreas. Quantitative RT-PCR detection of Wnt1, 2, 3, 3a, 4, 5a, 7a, 8a, 8b, 10b, 11 (C) and β-catenin and Dkk-1, 2, 3, 4 and sFRP-1, 2, 4, 5 (D) in normal controls (NC) and chronic pancreatitis. Data are presented as mean±SD from three independent experiments. *p<0.05 compared with NC.

**Figure 5:**
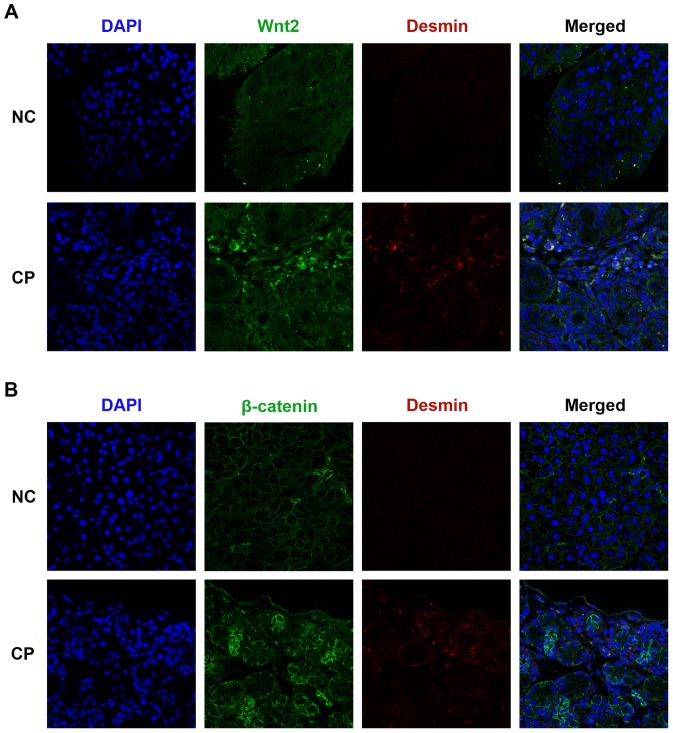
Immunofluorescent staining for Wnt2 and β-catenin *in vivo*. Double immunofluorescence staining for Wnt2 (green) (A) and β-catenin (green) (B) or desmin (red) (A, B) in the pancreas. DAPI (blue) was used to counterstain nuclei (magnification ×400). An overlap of Wnt2 or β-catenin and desmin in the pancreas of chronic pancreatitis was observed. Results from one representative mouse for each group are included.

Histopathological signs of CP such as abnormal architecture, glandular atrophy, pseudotubular complexes, fibrosis, and inflammatory cell infiltrates were observed in caerulein treated mice at the time of sacrifice (week 4) (Figure 4A). To determine whether the Wnt/β-catenin pathway is also activated in CP, double immunofluorescence for Wnt2 or β-catenin and desmin was performed. As shown in Figure 5A and 5B, the deposition of desmin was increased in the fibrotic tissue. In the normal pancreas β-catenin was observed surrounding acinar units. In CP mice, intense immunostaining for β-catenin was present in the fibrous septa and in the fibrotic stroma surrounding pancreatic acini, and the depositon of β-catenin was increased compared to that in normal tissues (Figure 5B). Wnt2 staining intensity was increased in residual acinar cells and PSCs in CP, particularly in the connective tissue surrounding fibrotic acini and at the interface between fibrotic septa and lobules (Figure 5A). A similar tendency was observed in the western blot analysis for Wnt2 and β-catenin, with an increase in nuclear β-catenin in CP mouse tissues (Figure 4B). Our qRT-PCR analysis revealed increased expression of Wnt2, β-catenin, sFRP1 and sFRP4, and decreased expression of Dkk-1 in CP mice, which was consistent with our observations in activated PSCs in vitro (Figure 4C, D). Taken together, these results suggested that the Wnt/β-catenin pathway was activated in CP.

### rmDkk-1 induces PSC apoptosis and inhibits PSC proliferation *in vitro*

**Figure 6:**
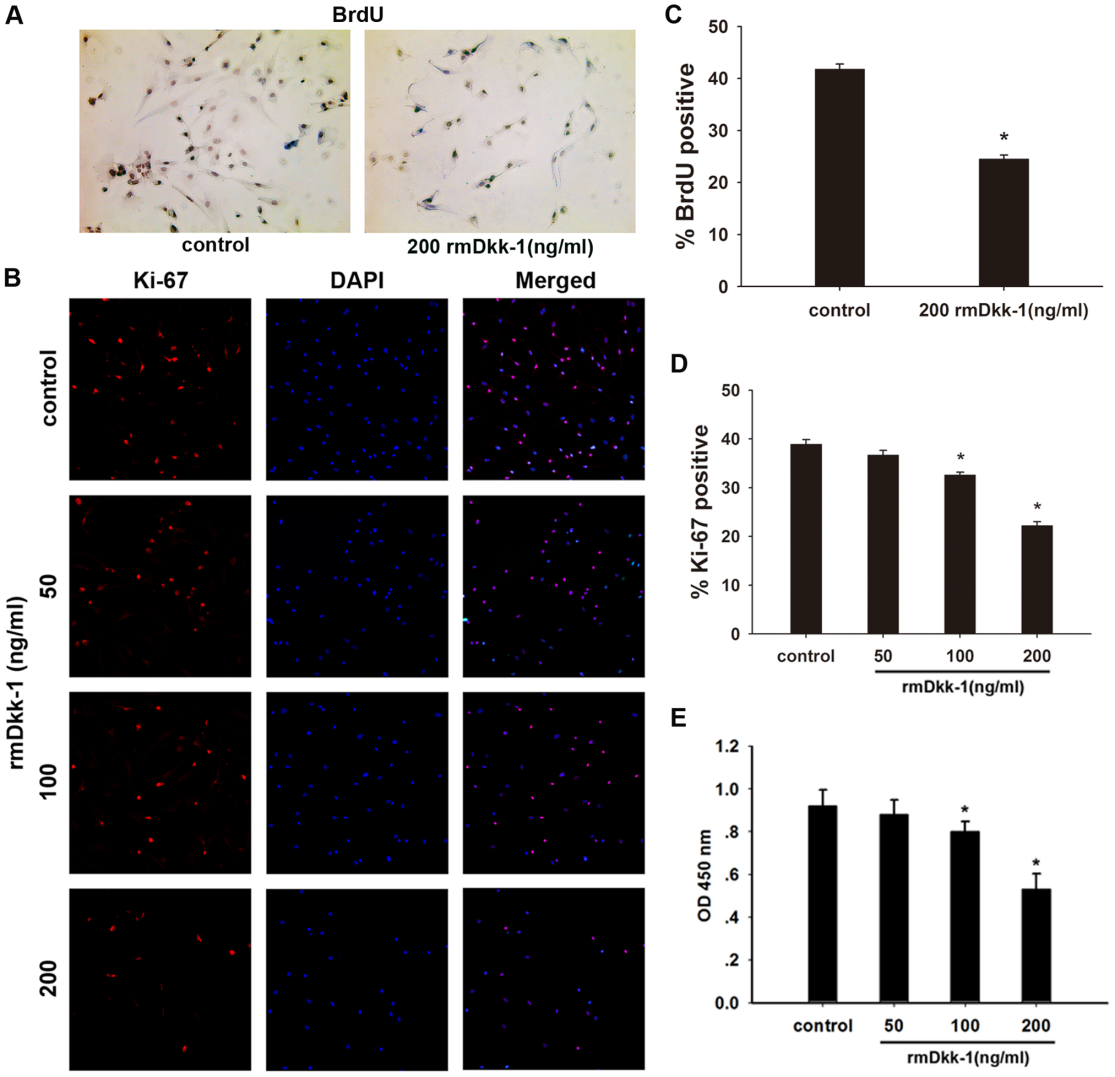
rmDkk-1 inhibits PSC proliferation *in vitro*. (A) BrdU incorporation. (B) Double immunofluorescence staining of Ki-67 and DAPI showed an overlapped distribution pattern of DAPI and Ki-67 confirming the anti-proliferative effect of rmDkk-1. Analysis of BrdU (C) and Ki-67 (D) staining. Data are presented as mean±SD from three independent experiments. *p<0.05 compared with the PBS-treated group. (E) One day PSCs were incubated with increasing doses of rmDkk-1 (0–200 ng/ml) for 72 h, and cell viability was assessed using the Cell Counting Kit-8 (CCK-8).

**Figure 7:**
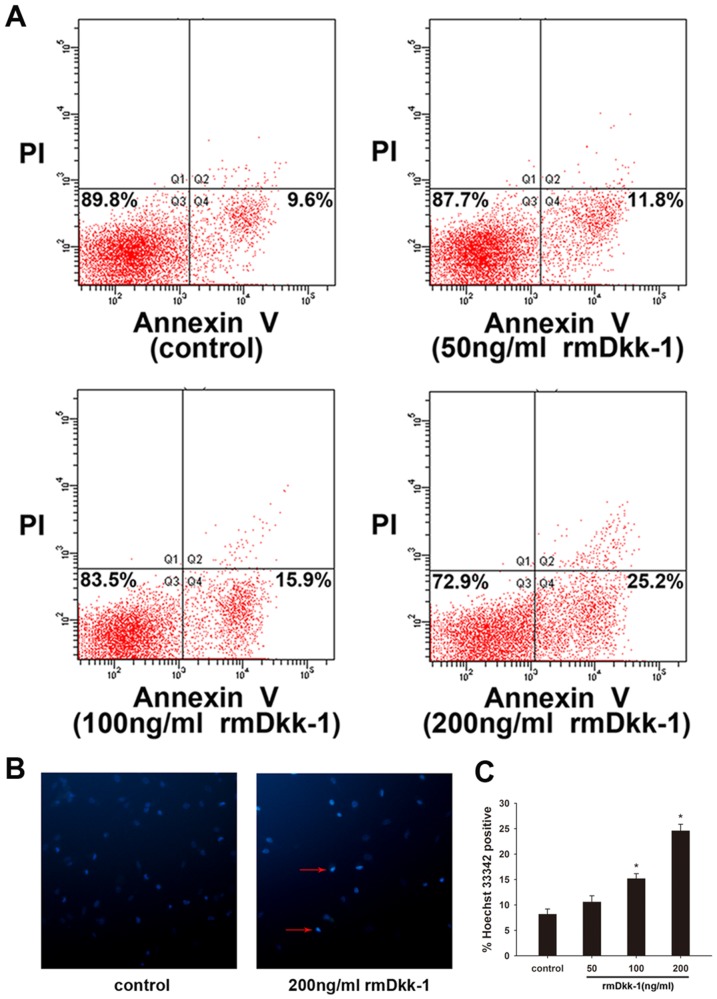
rmDkk-1 induces PSC apoptosis *in vitro*. (A) PSCs were labeled with Annexin V for 15 min at room temperature with 5 µg/ml PI. Apoptotic rates were compared among PSCs treated with or without rmDkk-1 at different doses by flow cytometry. (B) Hoechst 33342 staining. (C) Analysis of Hoechst 33342 staining. Data are presented as mean±SD from three independent experiments. *p<0.05 compared with the PBS-treated group.

Day 1 PSCs were incubated with or without rmDkk-1 at different doses (0, 50, 100, 200 ng/ml) as previously described. After day 5 PSCs were harvested and cell proliferation was determined by CCK-8 analysis, which showed that high doses of rmDkk-1 (200 ng/ml) inhibited the proliferation of PSCs (Figure 6E).Furthermore, assessment of Ki-67 expression in the PSC nucleus showed a significant reduction in the proliferation rate when the culture medium was supplemented with 200 ng/ml rmDkk-1 (Figure 6B, D). In addition, BrdU uptake was significantly decreased in cells treated with rmDkk-1 (200 ng/ml) compared to that in control cells (Figure 6A, C). These results indicated that treatment with high doses of rmDkk-1 inhibited the proliferation of PSCs. We then investigated whether the effect of rmDkk-1 was mediated by the induction of apoptosis in PSCs by annexin-V/PI staining and found that exposure to rmDkk-1 increased the rate of apoptosis, especially at a dose of 200 ng/ml (Figure 7A). In addition, quantification of cell apoptosis by Hoechst 33342 confirmed the induction of apoptosis by rmDkk-1 (200 ng/ml) (Figure 7B, C). These results indicated that a high dose of rmDkk-1 induced apoptosis and inhibited the proliferation of PSCs *in vitro*, resulting in a reduction of cell number.

### rmDkk-1 inhibits the activation of PSCs *in vitro*

**Figure 8:**
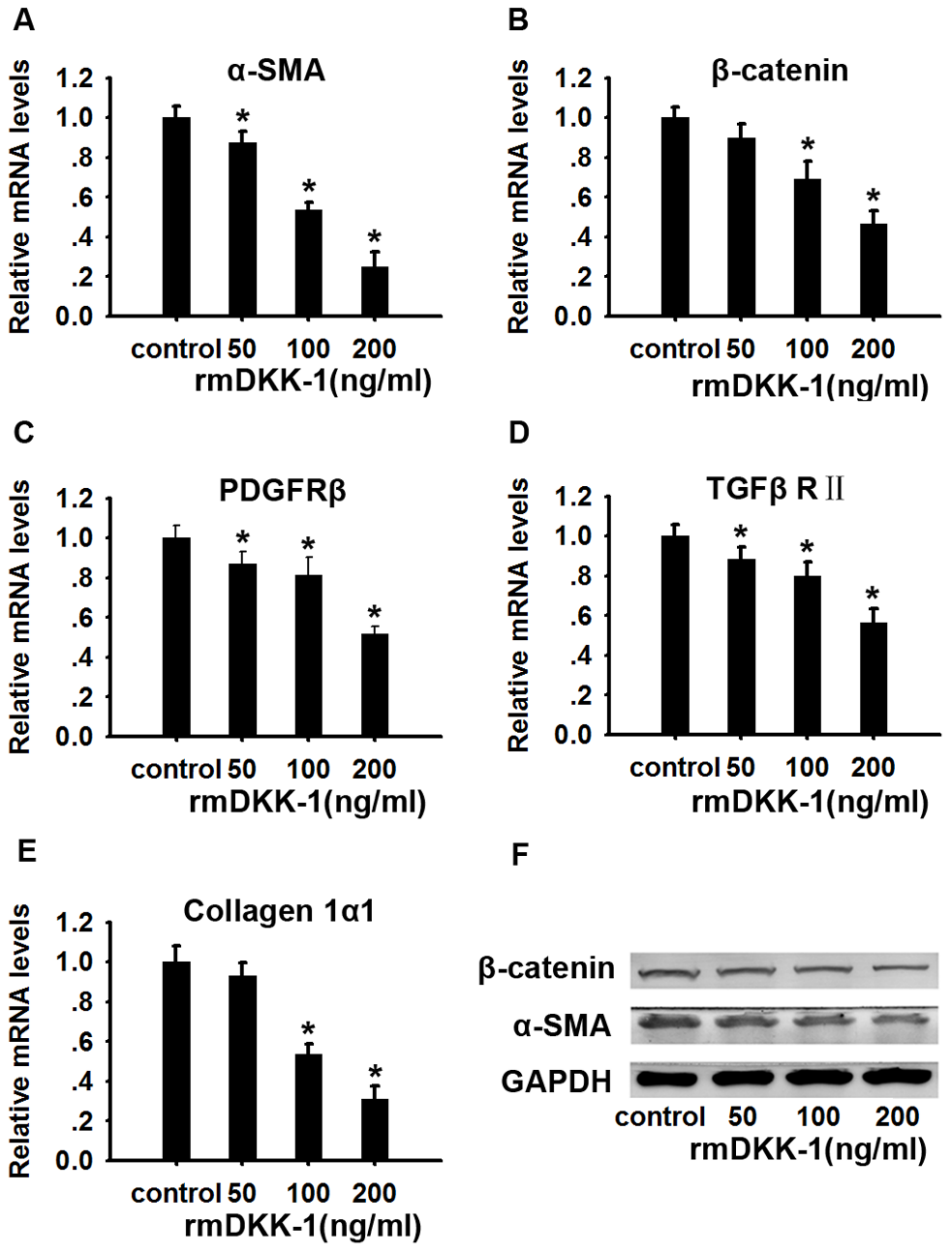
rmDkk-1 inhibits the activation of PSCs *in vitro*. Effect of rmDkk-1 on the activation of freshly isolated PSCs. PSCs isolated from mice were cultured for 24 hours and then incubated in fresh medium with or without rmDkk-1 at different doses. PSCs were cultured for 3 days and the mRNA levels of α-SMA (A), β-catenin (B), PDGFRβ(C), TGFβRII (D) and collagen1α1 (E) were analyzed by quantitative RT-PCR. GAPDH was used as a loading control. Data are presented as mean±SD from three independent experiments. *p<0.05 compared with the PBS-treated group. (F) Expression of β-catenin and α-SMA proteins in PSCs was analyzed by western blotting. GAPDH was used as a loading control.

Day 5 PSCs were harvested as previously described for quantitative RT-PCR and western blot analysis. Treatment with rmDkk-1 decreased β-catenin mRNA and protein levels in a concentration dependent manner, supporting our findings that the Wnt/β-catenin pathway was partly antagonized by rmDkk-1 (Figure 8B, F). α-SMA,a marker of PSC activation, was downregulated in response to rmDkk-1 treatment at both mRNA and protein levels, and it was especially inhibited by 200 ng/ml rmDkk-1 (Figure 8A, F). Blocking of the Wnt/β-catenin pathway inhibited the activation of PSCs, providing further evidence of the involvement of the Wnt/β-catenin pathway in activated PSCs. In addition, mRNA expression of collagen1α1, TGFβRII and PDGFRβ were persistently decreased in a dose-dependent manner (Figure 8C, D, E).

### rmDkk-1 inhibits the nuclear translocation of β-catenin in PSCs *in vitro*

**Figure 9:**
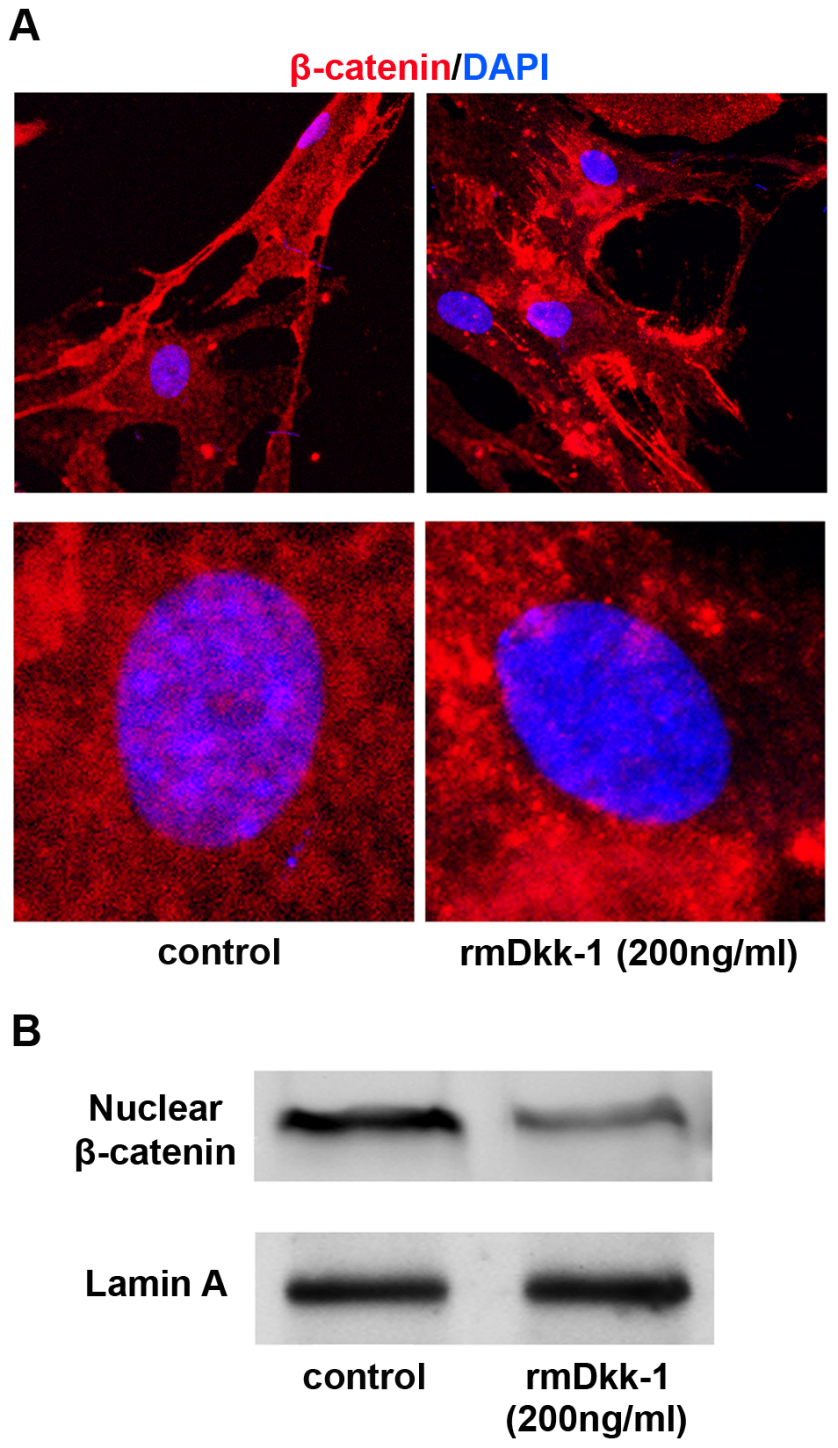
rmDkk-1 inhibits the nuclear translocation of β-catenin in PSCs *in vitro*. (A) Immunofluorescence staining of β-catenin (red) in PSCs. DAPI (blue) was used to counterstain nuclei. (B) Nuclear β-catenin protein levels were determined by western blotting. Lamin A was used as a loading control. All data are presented as the mean±SD of three independent experiments.

To determine whether rmDkk-1 inhibits the nuclear translocation of β-catenin, day 5 PSCs were harvested for immunofluorescence and western blot analysis. Administration of rmDkk-1 (200 ng/ml) for 3 days resulted in a marked decrease in nuclear β-catenin levels (Figure 9A), which further confirmed the inhibition of Wnt/β-catenin signaling by rmDkk-1. Consistent with these results, western blot analysis showed a significant downregulation of nuclear β-catenin levels in response to rmDkk-1 (200 ng/ml) (Figure 9B).

### Silencing of β-catenin inhibits the activation of PSCs *in vitro*

**Figure 10:**
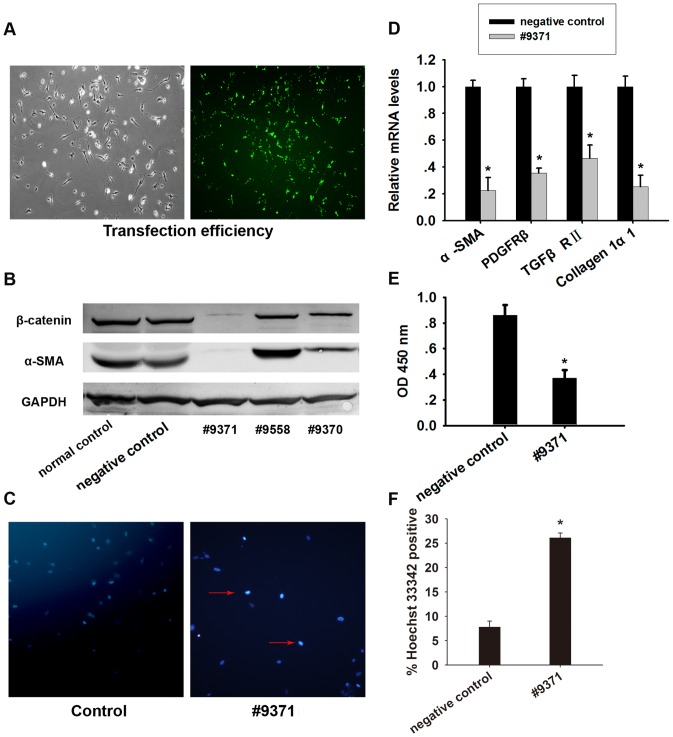
β-catenin-targeting siRNA inhibits the activation of PSCs *in vitro*. PSCs were transfected with negative control and β-catenin siRNA. (A) Transfection efficiency of PSCs. (B) β-catenin and α-SMA levels in cells were measured by western blotting. Blots were re-probed for GAPDH to confirm equal protein loading. (C) Hoechst 33342 staining. (D) The levels of α-SMA, PDGFRβ, TGFβRII and collagen1α1 mRNA were measured by quantitative RT-PCR. Data are presented as mean±SD from three independent experiments. *p<0.05 compared with negative control. (E) Cell viability was assessed using the Cell Counting Kit-8 (CCK-8). (F) Analysis of Hoechst 33342 staining. Data are presented as mean±SD from three independent experiments. *p<0.05 compared with negative control.

SiRNA mediated knockdown of β-catenin in day 1 PSCs and assessment of protein levels on day 5 showed a 90% inhibition of β-catenin expression (Figure 10A, B). Knockdown of β-catenin downregulated α-SMA expression at both mRNA and protein levels, suggesting that the activated PSCs partly restored the quiescent phenotype (Figure 10B, D). Moreover, the mRNA levels of collagen1α1, TGFβRII and PDGFRβ were remarkably reduced after siRNA transfection (Figure 10D). Knockdown of β-catenin led to an apparent reduction in cell number, which was further evaluated by Hoechst 33342 staining in day 5 PSCs. The results showed an increased rate of apoptosis in cells transfected with siRNA against β-catenin (Figure 10C, F). CCK-8 analysis showed that knockdown of β-catenin inhibited the proliferation of PSCs (Figure 10E). These results indicated that silencing β-catenin expression induced apoptosis and inhibited the proliferation of PSCs *in vitro*.

## Discussion

The Wnt signaling pathway plays an important role in a range of developmental and physiological processes including cell fate specification, tissue morphogenesis and homeostasis [28]. Its dysregulation has been associated with the development of fibrosis in the heart, kidney, liver and lung [19], [29]–[31]. However, little is known about the role of Wnt signaling in pancreatic fibrosis, a process in which PSCs play an important role. Therefore, in the present study, we firstly tested the role of Wnt signaling during the activation of PSCs *in vitro*.

Our results showed, for the first time, that Wnt/β-catenin signaling is activated during the activation of PSCs *in vitro*. The upregulated genes included Wnt1, 2, 3a and 10b and β–catenin, whereas Dkk-1, 2, 3 and 4 were significantly downregulated. Our screen for Wnts upregulated following the activation of PSCs showed that Wnt2 was the only Wnt that was significantly upregulated after the activation of PSCs. Furthermore, we also found that the levels of Wnt2 and β-catenin proteins were significantly increased in the pancreas of caerulein-induced CP mice. Duan et al reported that a pro-fibrotic Wnt1/β-catenin injury response is critically required for preserving cardiac function after acute ischemic cardiac injury [29]. Wnt4 is known to contribute to renal fibrosis [14], and Wnt3a and Wnt5a are involved in lung and liver fibrosis respectively. These data indicate that Wnt ligands elicit profibrotic effects in a tissue-specific manner in different organs.

Activation of the canonical Wnt/β-catenin pathway results in the accumulation of β-catenin in the cytoplasm and its eventual translocation into the nucleus to act as a transcriptional coactivator of transcription factors belonging to the T cell factor/lymphocyte enhancer factor (TCF/LEF) family [8]. Therefore, we detected the nuclear translocation of β-catenin in activated PSCs, and found that β-catenin localized to the nucleus in activated PSCs *in vitro* and in a mouse model of CP. Our results suggested that the Wnt/β-catenin pathway was activated during the activation of PSCs. The activation of Wnt signaling in PSCs and in an animal model of pancreatic fibrosis has not been described previously, and it suggests an important role for Wnt2/β-catenin signaling in the development of pancreatic fibrosis.

Wnt signals are negatively regulated by secreted antagonists that bind directly to the ligand, such as the secreted frizzled-related protein (sFRP) family, or that prevent LRP coreceptor association with Fz. Dickkopf (Dkk), the best-characterized of the latter group of antagonists, binds to LRP6 and inhibits the formation of the Wnt-induced Fz-LRP5/6 complex that is essential to the canonical Wnt/β-catenin pathway [8]. Previous studies have shown the repressive function of sFRP4 in Wnt signaling [32], [33]. Bayle et al reported the upregulation of sFRP4 in response to increased Wnt2 in Tsk mouse skin, which indicated that sFRP4 might antagonize upregulated Wnt expression [18]. Froeling et al showed that human primary and metastatic pancreatic tumor tissues stained strongly for cancer cell nuclear β-catenin whereas low levels of sFRP4 were present in cancer cells and PSCs [4]. In the present study, we found that both Wnt2 and sFRP4 were markedly increased in fully activated PSCs, suggesting the existence of other regulatory mechanisms for sFRP4 in PSCs. Future studies are needed to clarify the mechanisms regulating sFRP4 and its role in CP and pancreatic cancer.

Although Dkk-1 expression in CP tissues is difficult to detect by western blotting (data not shown), we showed that Dkk-1 levels were decreased during the activation of PSCs *in vitro*. Dkk-1 is a member of the Dickkopf family of secreted proteins, which play a significant role as negative regulators of the canonical Wnt/β-catenin pathway. Dkk-1 binds to low-density lipoprotein receptor-related protein (LRP) 5/6 with high affinity. In addition, Dkk-1 binds to another class of receptors, Kremen1 and 2 (Krm1/2). Dkk-1 can form a ternary complex with Krm2 and LRP6 and induce rapid endocytosis and removal of LRP6 from the plasma membrane [13], [34]. Dkk-1 is upregulated in response to Wnt signaling activation, initiating a negative feedback loop [35], [36]. Previous studies have shown that Dkk-1 expression is downregulated under certain circumstances. In certain tumor cells, the epigenetic silencing of Dkk-1 by promoter hypermethylation contributes to aberrant Wnt/β-catenin signaling [37], [38]. In addition, the glycosylation of secreted Dkk-1 has been suggested [39]. Further work is required to clarify the molecular mechanism underlying the deregulation of Dkk-1 and aberrant Wnt/β-catenin signaling associated with the activation of PSCs.

In the present study, we hypothesized that an imbalance of the Wnt/Dkk negative feedback loop may promote the persistent activation of PSCs. We therefore corrected the imbalance of Wnt/Dkk negative feedback by restoring the level of Dkk-1 using rmDkk-1, which resulted in the partial inhibition of the Wnt signaling pathway. In addition, rmDkk-1 inhibited PSC activation and collagen synthesis, and high levels of rmDkk-1 expression inhibited proliferation and induced apoptosis in PSCs. Inhibition of Wnt signaling inhibited PSC activation *in vitro*. Knockdown of β-catenin and restoration of Dkk-1 expression downregulated the expression of α-SMA, collagen1α1, TGFβRII and PDGFRβ, inhibited proliferation and induced apoptosis. These results provide further evidence of the important role of the Wnt/β-catenin pathway in PSC activation and shed light on the molecular mechanisms of PSC activation.

In the present study, treatment of PSCs with the Wnt antagonist Dkk-1 inhibited their activation and attenuated canonical Wnt/β-catenin signaling. The β-catenin-dependent pathway, which is triggered by the interaction of Wnt with Frizzled and LRP5 or LRP6, is prominently involved in the regulation of cell differentiation and proliferation [40]. This may explain why a high level of Dkk-1 inhibited proliferation and induced apoptosis in PSCs. In addition, rmDkk-1 may act by correcting the imbalance in the Wnt/Dkk negative feedback loop, thus inhibiting the persistent activation of PSCs. The present study did not test the therapeutic efficacy of Dkk-1 in CP. This therapeutic approach will be best implemented by expressing Dkk-1 in a manner that specifically targets activated PSCs *in vivo*. β-catenin may be involved in pancreatic repair in CP, which is particularly important because global Dkk-1 expression may inhibit pancreatic regeneration requiring canonical Wnt signaling [41].

In conclusion, we showed that the Wnt2/β-catenin pathway was activated during the activation of PSCs *in vivo* and *in vitro*. Our data suggested that an imbalance in Wnt/Dkk negative feedback signaling promotes the persistent activation of PSCs in CP, although the exact underlying mechanism remains unclear. We showed that Dkk inhibited the proliferation and profibrotic phenotype of PSCs by downregulating the expression of collagen1α1, TGFβRII and PDGFRβ via a block of the Wnt/β-catenin pathway. These results indicate that targeting Wnt2/β-catenin/Dkk1 may be a promising therapeutic strategy for the treatment of CP.
